# Experimental data on the shear resistance of headed studs welded within the ribs of profiled steel decking transverse to the supporting beams

**DOI:** 10.1016/j.dib.2023.109616

**Published:** 2023-09-23

**Authors:** Stephen J. Hicks, Vitaliy V. Degtyarev

**Affiliations:** a School of Engineering, University of Warwick, Coventry CV4 7AL, United Kingdom; b New Millennium Building Systems, LLC, 3700 Forest Dr. Suite 501, Columbia, SC 29204, United States

**Keywords:** Headed studs, Shear resistance, Profiled steel sheeting, Composite steel and concrete structures, Composite slabs, Machine learning, Push-out tests, Composite steel and concrete beams

## Abstract

This article describes a dataset with 464 push test results for studs welded within the ribs of profiled steel decking transverse to the supporting beams. The experimental data were collected from 30 publications dated from 1980 to 2017. The dataset presents the measured shear resistance per stud, with over 20 nominal or measured parameters, including: the properties of studs, deck, and concrete; the number of studs within a concrete rib; and the dimensions determining stud position within the concrete rib. This article presents and discusses the statistical parameters of the dataset variables, their distributions, and correlations. The dataset supports the identification of the key design variables that affect the stud shear resistance. It also provides information for evaluating the accuracy and reliability of existing design models, and may be used to form the basis for developing new predictive models.

Specifications Table

**Table:** 

Subject	Civil and Structural Engineering
Specific subject area	Composite Steel and Concrete Structures
Data format	Raw
Type of data	Table
Data collection	The experimental data were collected from the literature, with all sources referenced.
Data source location	Bibliographic references are provided within the database spreadsheet.
Data accessibility	Repository name: Mendeley Data Data identification number: DOI: 10.17632/nfmhnzbfy9.2 Direct URL to data: https://data.mendeley.com/datasets/nfmhnzbfy9 Instructions for accessing these data: N/A
Related research article	V.V. Degtyarev, S.J. Hicks, Shear resistance of welded studs in deck slab ribs transverse to beams, Engineering Structures. 294 (2023) 116709. https://doi.org/10.1016/j.engstruct.2023.116709

## 1 Value of the Data

• The resistance of composite beams in steel-framed buildings depends on the longitudinal shear transfer between the steel beam and the concrete slab through the provision of shear connectors. The most widely used type of shear connector is the headed stud because: the drawn-arc stud welding process is rapid; they provide little obstruction to reinforcement within the concrete slab; and they are equally strong and stiff in shear in all directions normal to the axis of the stud. When studs are welded in steel decking with the ribs transverse to the supporting beams, the shear resistance is reduced.
• To account for the presence of the deck slab rib, the resistance has traditionally been determined in many international design standards by multiplying the resistance of a stud embedded within a solid concrete slab by a reduction factor, which has been determined from push tests and accounts for the geometry of the deck in relation to the stud. The existing empirical design models for the stud resistance in deck slabs were developed considering smaller datasets than that presented in the present articule, and do not always produce accurate and safe results.
• The presented data is useful for identifying design variables affecting the stud shear resistance, evaluating the performance of existing design models, and developing new predictive models, as described in [1,2] and implemented in a web application at https://studs-in-deck-slabs.herokuapp.com.
• Researchers and practitioners working in the field of composite steel and concrete structures can benefit from the data; in particular, those working on improving design models for stud resistance, and developing national and international design standards.
• The data will assist in the identification of underrepresented design parameters, which will be beneficial in extending knowledge in future experimental investigations, and improving design models in terms of their accuracy and reliability.

## 2 Data Description

The dataset [3] presents the measured shear resistance per stud from 464 push tests, along with over 20 parameters featured in the test specimens, including: the mechanical properties of the studs, deck, and concrete; the number of studs within a concrete rib, the stud welding technique, and the number of ribs; the geometry defining the stud position within the concrete rib, and the position of the reinforcement bars; and the level of normal force applied to the face of the test slabs. Nominal values of the parameters required for reliability analyses in accordance with Eurocodes are also presented.

The dataset is presented within a Microsoft Excel file in XLSX format with four worksheets. The first worksheet, entitled “Database,” contains the dataset. The second worksheet, “Notes,” provides bibliographic references to the publications used for the dataset collection. The third worksheet, “Diagrams,” presents figures identifying the geometric variables used to describe the deck cross-section, welded stud connectors and the positions of the studs used in the dataset. Finally, the fourth worksheet contains a quality record.

The dataset includes the mean measured shear resistance per stud, *P*_em_, and the following 28 measured parameters:

• the normal force on the face of the test slab as a percentage of longitudinal force, *N*_norm_;
• the number of ribs per test slab, *N*_ribs_;
• the number of studs per rib, *n*_r_;
• stud position: favorable (F), central (C), unfavorable (U), or staggered (S);
• stud welding: through-deck, or holes;
• location of reinforcement measured downwards from the top of the stud, *z*_s_;
• overall slab thickness including decking, *h*_cs_;
• width of test slab, *b*_slab_;
• deck name;
• the thickness of the deck, *t*;
• deck type: trapezoidal, or re-entrant;
• width of the top of the concrete rib, *b*_top_;
• width of the bottom of the concrete rib, *b*_bot_;
• concrete rib width, measured at mid-height for a trapezoidal deck or at the top for a re-entrant deck, *b*_0_;
• net depth of decking excluding longitudinal stiffener on the crest, *h*_pn_;
• gross depth of decking including longitudinal stiffener on the crest, *h*_pg_;
• transverse spacing between studs within rib, *s*_y_;
• longitudinal spacing between studs within rib, *s*_x_;
• longitudinal distance from the stud centre to the deck web at the trapezoidal deck mid-height or the re-entrant deck top, *e*;
• longitudinal distance from the rib top corner to the nearest stud centre, *e*_t_;
• concrete compressive strength, *f*_cm_;
• concrete secant modulus of elasticity, *E*_cm_;
• ultimate tensile strength of studs, *f*_um_;
• yield strength of decking, *f*_ypm_;
• diameter of stud shank, *d*_m_;
• weld collar diameter, *d*_dom_;
• weld collar height, *h*_wm_;
• stud height after welding, *h*_scm_; and
• the stud height-to-diameter ratio, *h*_scm_/*d*_m_.

The following nominal properties required for reliability analyses in accordance with the Eurocodes are also presented in the dataset:

• the characteristic compressive cylinder strength, *f*_ck_, in accordance with EN 1992-1-1 [4];
• concrete secant modulus of elasticity, *E*_cm_, calculated from *f*_ck_ in accordance with EN 1992-1-1 [4];
• ultimate tensile strength of studs, *f*_u_, in accordance with EN ISO 13918 [5];
• yield strength of decking, *f*_yp_;
• stud shank diameter, *d*;
• weld collar diameter, *d*_do_;
• weld collar height, *h*_w_;
• the stud height-to-diameter ratio, *h*_sc_/*d*; and
• specified as-welded stud height, *h*_sc_.

The measured concrete compressive strength, *f*_cm_, was reported in the publications for different cube and cylinder specimens cured at different conditions. It was converted into the cylinder strength as described in [6]. When the measured concrete secant modulus of elasticity, *E*_cm_, was not reported, it was computed based on *f*_cm_ in accordance with the fib Model Code 2010 [7]. Unless measured values were provided, the weld collar diameter, *d*_dom_, and height, *h*_wm_, were taken from EN ISO 13918 [5]. Finally, unless the specified concrete strength was reported, the characteristic compressive cylinder strength *f*_ck_ according to the strength classes given in EN 206 [8] was evaluated using the comparison of means method from ISO 12491 [9].

**Fig. 1:**
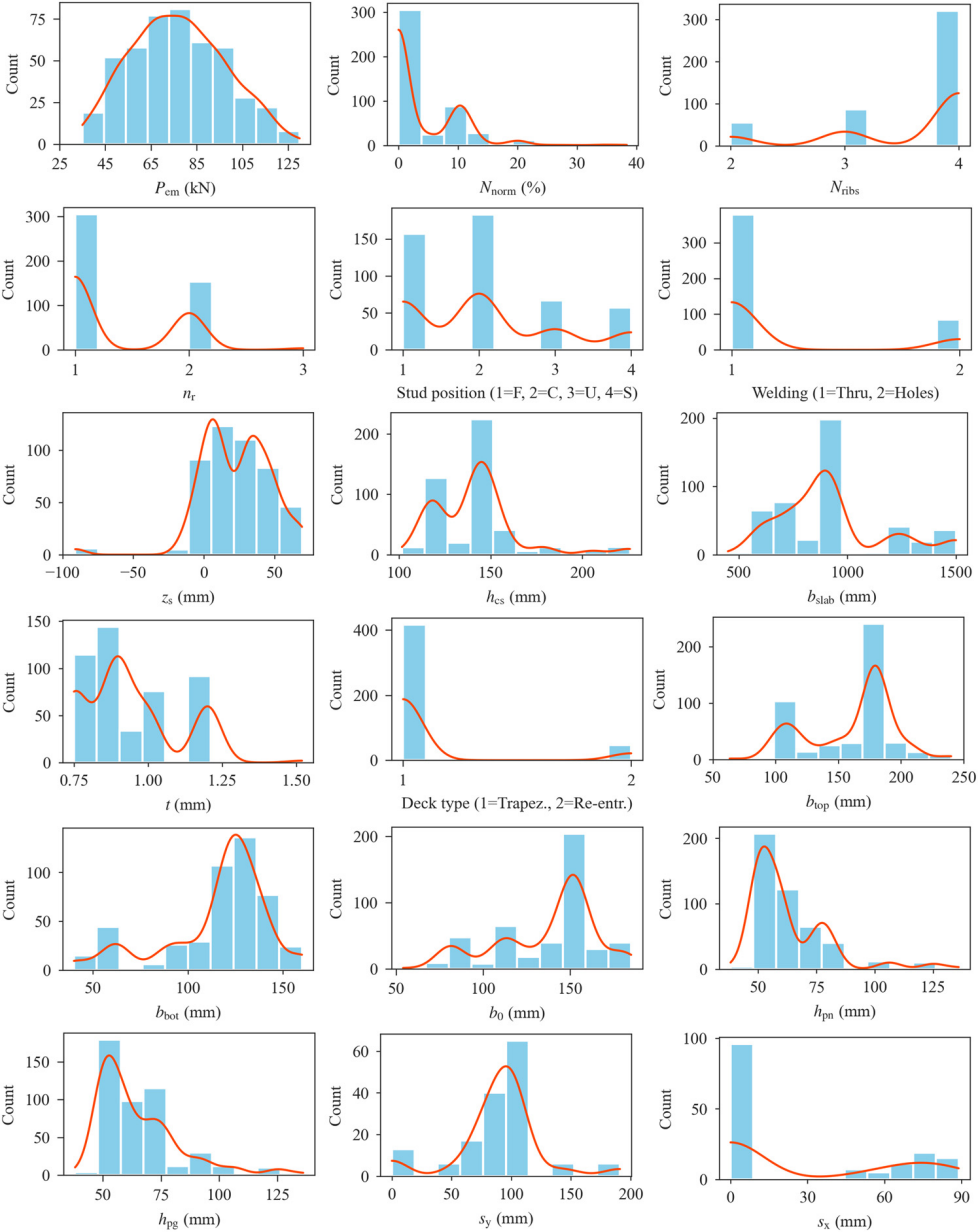

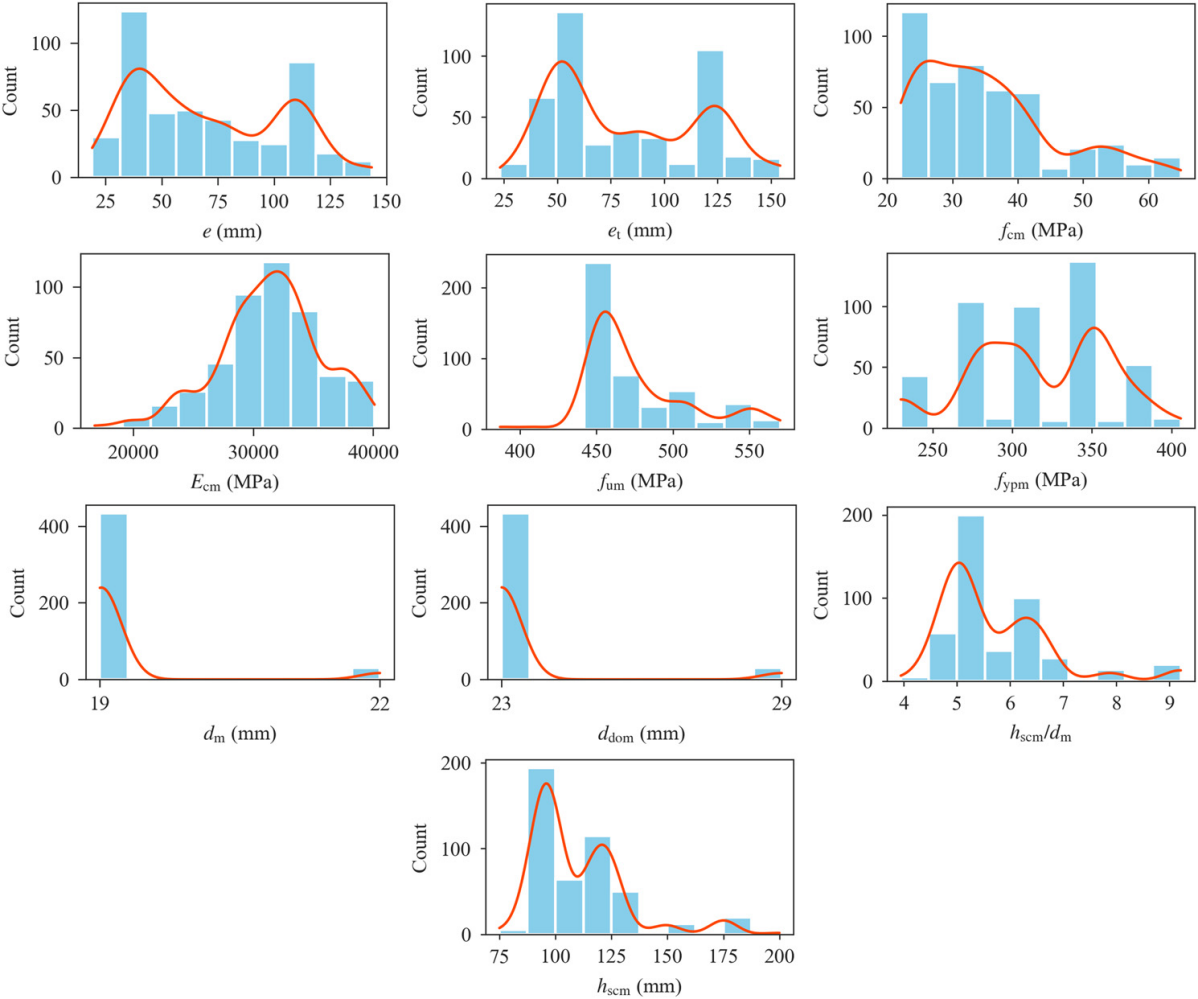
Distributions of database variables.

**Fig. 2:**
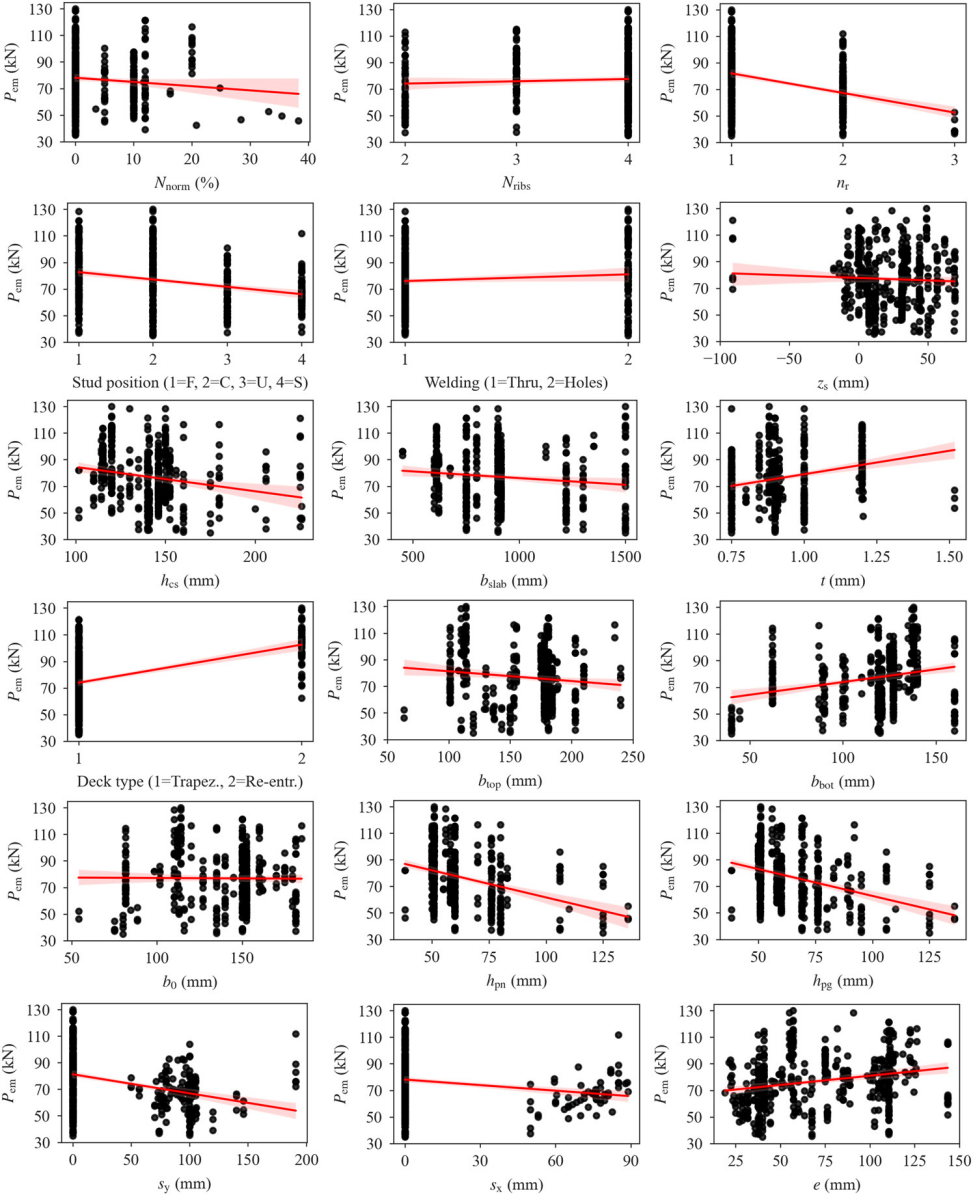

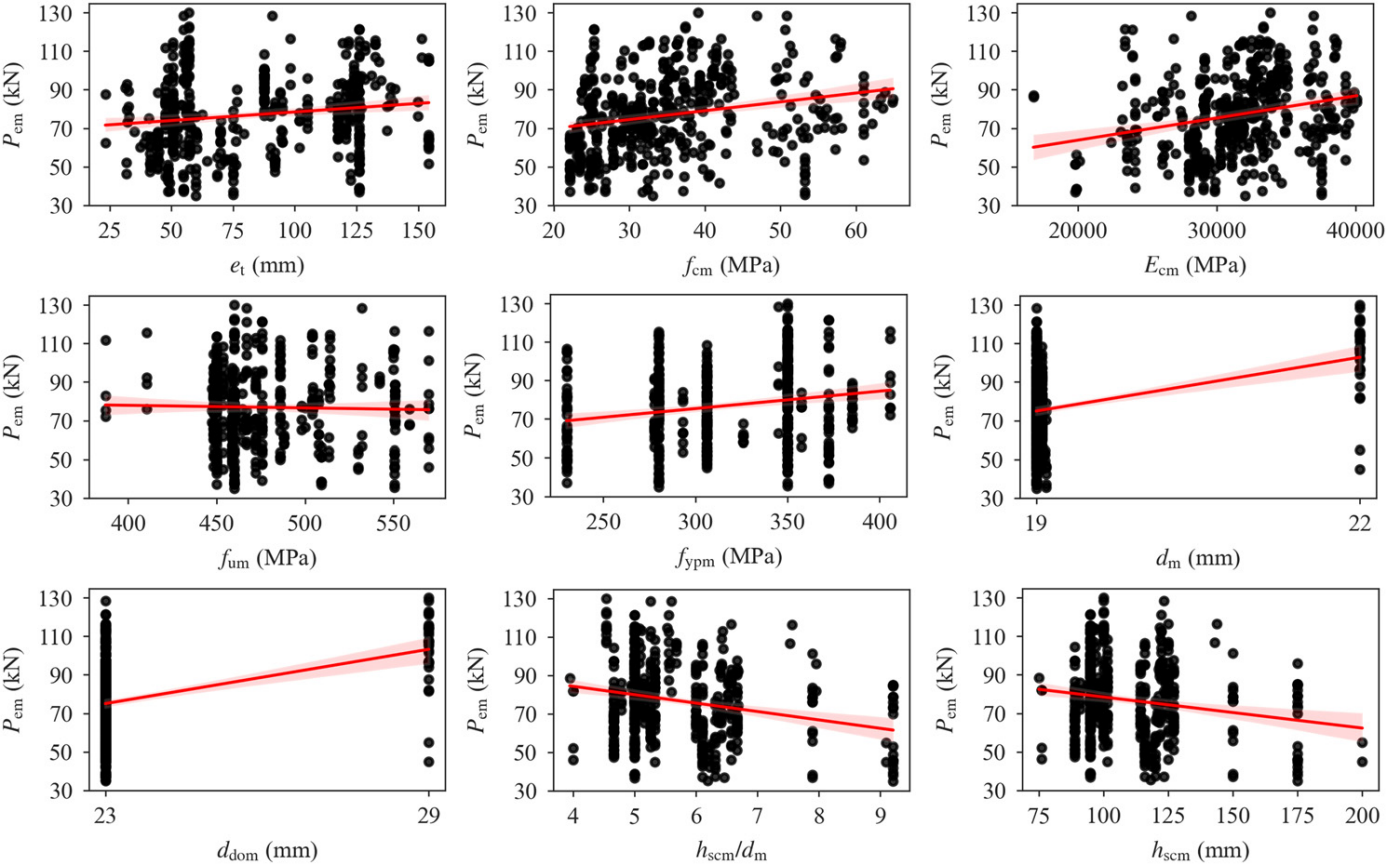
Stud shear resistance as a function of dataset variables.

Table 1 presents the statistical parameters of the database variables. Fig. 1 shows distributions of the database variables. Regression plots of stud shear resistance as a function of dataset variables are given in Fig. 2, with the red lines indicating the linear regression lines. The shaded regions around the regression lines indicate 95% confidence intervals. A pairwise Pearson correlation coefficient matrix of the database variables is also presented in Table 2.

**Table 1:** Statistical parameters of the database variables.

Variable	Min	Max	Mean	Standard Deviation	Skewness	Kurtosis
*P*_em_ (kN)	35.00	130.00	76.87	20.36	0.19	−0.58
*N*_norm_ (%)	0.00	38.35	3.82	6.11	1.87	4.73
*N*_ribs_	2.00	4.00	3.57	0.70	−1.33	0.31
*n*_r_	1.00	3.00	1.36	0.51	0.91	−0.50
Stud position	1.00	4.00	2.05	0.99	0.67	−0.56
Welding	1.00	2.00	1.18	0.39	1.64	0.70
*z*_s_ (mm)	−91.00	69.34	23.01	25.10	−0.93	3.77
*h*_cs_ (mm)	101.60	226.00	141.50	23.35	1.51	3.50
*b*_slab_ (mm)	450.00	1500.00	923.16	247.40	0.91	0.24
*t* (mm)	0.75	1.52	0.94	0.16	0.73	−0.09
Deck type	1.00	2.00	1.10	0.30	2.65	5.05
*b*_top_ (mm)	63.50	240.00	160.99	34.54	−0.54	−0.58
*b*_bot_ (mm)	40.00	160.00	115.63	27.36	−1.12	0.79
*b*_0_ (mm)	53.98	185.00	137.03	28.68	−0.74	−0.27
*h*_pn_ (mm)	38.10	136.00	62.66	17.14	2.00	4.50
*h*_pg_ (mm)	38.10	136.00	65.10	17.94	1.60	2.69
*s*_y_ (mm)	0.00	191.00	30.60	47.47	1.18	0.22
*s*_x_ (mm)	0.00	88.90	8.83	23.95	2.42	4.05
*e* (mm)	19.05	143.50	70.47	32.82	0.39	−1.13
*e*_t_ (mm)	23.25	154.25	82.42	34.48	0.40	−1.24
*f*_cm_ (MPa)	22.10	64.94	35.21	10.38	0.93	0.16
*E*_cm_ (MPa)	16789.40	40112.00	31383.47	4257.58	−0.37	0.34
*f*_um_ (MPa)	387.10	570.00	476.47	35.03	1.03	0.64
*f*_ypm_ (MPa)	230.00	405.80	316.37	44.03	−0.25	−0.67
*d*_m_ (mm)	19.00	22.00	19.21	0.73	3.55	10.63
*d*_dom_ (mm)	23.00	29.00	23.39	1.48	3.55	10.66
*h*_wm_ (mm)	6.00	6.00	6.00	0.00	0.00	0.00
*h*_scm_/*d*_m_	3.95	9.21	5.72	1.08	1.55	2.55
*h*_scm_ (mm)	75.00	200.00	109.88	21.15	1.62	3.02

**Table 2:** Correlation matrix of database variables.

## 3 Experimental Design, Materials and Methods

The dataset was collected from 30 publications, dating from 1980 to 2017, which describe push tests on studs welded within the ribs of profiled steel decking transverse to the supporting beams. The publications included in the database were identified through an extensive literature survey, and included tests that were considered in the original calibration of Eurocode 4 [10,11]. The following criteria for the selection of the test data was used: sufficient measurements of the material strengths and geometry of the push tests were reported in the publications; information on the geometry defining the stud position within the concrete rib, the position of the reinforcement bars, and the level of normal force applied to the face of the specimens was available; and no indication of poor stud welding, low concrete strength, or unexpected problems were reported. Details on the design of the test specimens, materials, and methods used in each test programme can be found in the publications referenced in the database.

## 4 Limitations

None.

## Ethics Statement

The authors declare that they have followed the general ethics rules of scientific research performance and publishing. This work did not involve human subjects, animal experiments, or data collected from social media platforms.

## CRediT authorship contribution statement

**Stephen J. Hicks:** Conceptualization, Methodology, Validation, Investigation, Data curation, Writing – review & editing, Supervision. **Vitaliy V. Degtyarev:** Conceptualization, Methodology, Software, Validation, Formal analysis, Investigation, Writing – original draft, Writing – review & editing, Visualization.

## Data Availability

Database of push tests on specimens with headed stud shear connectors welded within the ribs of profiled steel decking transverse to the supporting beams (Original data) (Mendeley Data).
